# Association between systemic lupus erythematosus and inflammatory bowel disease in European and East Asian populations: a two-sample Mendelian randomization study

**DOI:** 10.3389/fimmu.2023.1199896

**Published:** 2023-11-03

**Authors:** Weidong Xie, Haojie Jiang, Yao Chen, Huanhao Zhang, Yaoyu Song, Zhaojie Yu, Huayan Gu, Hongkai Xu, Saiyi Han, Sen Li, Naxin Liu, Shaoliang Han

**Affiliations:** ^1^ Department of The Gastrointestinal Surgery, First Affiliated Hospital of Wenzhou Medical University, Wenzhou, China; ^2^ Department of Medical Oncology, Sir Run Run Shaw Hospital, School of Medicine, Graduate School, Zhejiang University, Hangzhou, China; ^3^ Wenzhou Medical University, Wenzhou, China; ^4^ Department of Breast Surgery, First Affiliated Hospital of Wenzhou Medical University, Wenzhou, China; ^5^ The Quzhou Affiliated Hospital of Wenzhou Medical University, Quzhou people’s Hospital, Quzhou, China; ^6^ School of Basic Medical Sciences, Wenzhou Medical University, Wenzhou, China

**Keywords:** Mendelian randomization (MR), systemic lupus erythematosus (SLE), inflammatory bowel disease (IBD), ulcerative colitis (UC), Crohn’s-disease (CD)

## Abstract

**Background:**

Previous studies have shown a coexistence phenomenon between systemic lupus erythematosus (SLE) and inflammatory bowel disease (IBD), but the causal relationship between them is still unclear. Therefore, we conducted a two-sample Mendelian randomization (MR) analysis using publicly available summary statistics data to evaluate whether there was a causal relationship between the two diseases.

**Methods:**

Summary statistics for SLE and IBD were downloaded from the Open Genome-Wide Association Study and the International Inflammatory Bowel Disease Genetics Consortium. European and East Asian populations were included in this MR work. We adopted a series of methods to select instrumental variables that are closely related to SLE and IBD. To make the conclusion more reliable, we applied a variety of different analysis methods, among which the inverse variance–weighted (IVW) method was the main method. In addition, heterogeneity, pleiotropy, and sensitivity were assessed to make the conclusions more convincing.

**Results:**

In the European population, a negative causal relationship was observed between SLE and overall IBD (OR = 0.94; 95% CI = 0.90, 0.98; P < 0.004) and ulcerative colitis (UC) (OR = 0.93; 95% CI = 0.88, 0.98; P = 0.006). After removing outliers with Mendelian Randomization Pleiotropy RESidual Sum and Outlier (MR-PRESSO), the results remained consistent with IVW. However, there was no causal relationship between SLE and Crohn’s disease. In the East Asian population, no causal relationship was found between SLE and IBD.

**Conclusion:**

Our results found that genetic susceptibility to SLE was associated with lower overall IBD risk and UC risk in European populations. In contrast, no association between SLE and IBD was found in East Asian populations. This work might enrich the previous research results, and it may provide some references for research in the future.

## Introduction

Inflammatory bowel disease (IBD) is a chronic idiopathic gastrointestinal disorder that includes Crohn’s disease (CD) and ulcerative colitis (UC) (1). Although a great deal of research has been done on IBD over a long period of time, the relationship between genetic susceptibility, environmental and other factors, and IBD is still controversial (2). Although IBD was previously considered a Western disease, the incidence of the disease has increased rapidly in Asia and other parts of the world in recent years (3). Over the past decade, IBD has become a huge burden on global public health (4). As a result, attempts have been made to clarify the risk and protective factors of IBD to better understand and prevent the disease.

Systemic lupus erythematosus (SLE) is a chronic autoimmune disease that can involve multiple organs and tissues throughout the body, such as the skin and kidneys (5). The pathogenesis of SLE is complex, and the interplay of genetic and environmental factors is pointed out to be the cause of SLE (6). Although SLE and IBD seem to be two unrelated diseases, it has been found that some patients with SLE have IBD in combination (7), which seems to hint that there may be a link between the two diseases. Therefore, it is necessary to explore the relationship between the SLE and IBD further.

In this study, we conducted a Mendelian randomization (MR) study to further confirm the causal relationship between SLE and IBD. MR is an epidemiological analysis method that assesses the causal relationship between exposure and outcome by using single-nucleotide polymorphisms (SNPs) as instrumental variables (IVs) for exposure (8). The method avoids irrelevant confounders, such as environmental exposures, and it reduces the effect of reverse causality, thus making the results more convincing (9).

## Methods

### Study design

**Figure 1:**
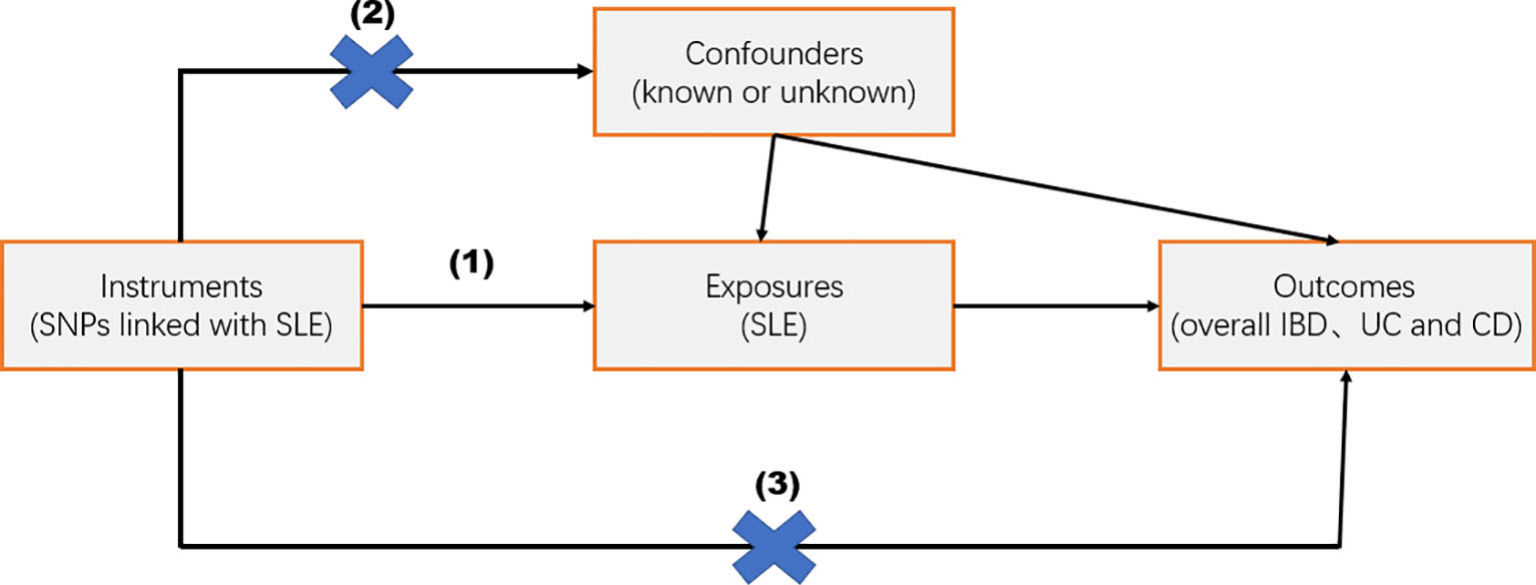
Overview of the study design. Mendelian randomization studies were based on three assumptions: (1) the instrumental variable (IV) was strongly related to exposure; (2) IV was independent of known or unknown confounding factors; and (3) IV affected the outcome only through exposure factors.

MR analyses need to fulfill the following three assumptions: (1) the IV is strongly associated with exposure; (2) the IV is not associated with any confounders affecting the exposure–outcome association; and (3) the IV affects the outcome only through exposure (**Figure 1**). It is important to note that the data used in this study are publicly available and free of charge, so there is no need to provide further ethical review and informed consent again.

### Data sources

SLE-associated SNPs in European populations were extracted from a large-scale Genome-Wide Association Study (GWAS) study that included 5,201 cases and 9,066 controls (10). The IBD-associated GWAS data for the European population were obtained from the International Inflammatory Bowel Disease Genetics Consortium (IIBDGC), which was the world’s largest genetic database for IBD. The overall IBD-associated GWAS data for the European population contains 31,665 cases and 33,977 controls, UC for the European population contains 13,768 cases and 33,977 controls, and CD for the European population contains 17,897 cases and 33,977 controls (11). In addition, the SLE-associated SNPs in the East Asian population were derived from a large GWAS study containing 4,222 cases and 8,431 controls (12). Genetic data related to IBD in East Asian populations were also obtained from IIBDGC, with overall IBD in East Asian populations containing 2,824 cases and 3,719 controls, UC in East Asian populations containing 1,134 cases and 3,719 controls, and CD in East Asian populations containing 1,690 cases and 3,719 controls (11). The status of each data is listed in **Table 1**. These data are publicly available and can be accessed at https://gwas.mrcieu.ac.uk/ and www.ibdgenetics.org.

**Table 1:** Data sources.

Phenotype	Data source	Sample size (cases/control)	Ancestor
Exposure			
SLE	Bentham et al.	5,201/9,066	European
SLE	Wang YF et al.	4,222/8,431	East Asian
Outcome			
IBD	IIBDGC	31,665/33,977	European
IBD	IIBDGC	2,824/3,719	East Asian
UC	IIBDGC	13,768/33,977	European
UC	IIBDGC	1,134/3,719	East Asian
CD	IIBDGC	17,897/33,977	European
CD	IIBDGC	1,690/3,719	East Asian

SLE, systemic lupus erythematosus; IBD, inflammatory bowel disease; UC, ulcerative colitis; CD, Crohn’s disease; IIBDGC, International Inflammatory Bowel Disease Genetic Consortium.

### Selection of genetic instruments

In this study, we selected IVs on the basis of the following criteria (13): (1) SNPs were strongly associated with SLE, so P < 5 × 10^−8^ was used as the primary screening condition; (2) SNPs were independent of confounding factors affecting SLE and IBD, and, to ensure that exposure-related IVs were independent, we excluded SNPs with linkage disequilibrium (R^2^ < 0.001, clumped = 10,000 kb). (3) SNPs were not directly associated with IBD and could only act on IBD through SLE.

In addition, we calculated the F-statistic to eliminate the bias in the results caused by weak IVs. The F-statistic was calculated as F = β2/se2, and the F-statistic was required to be >10 (14).

### Statistical analysis

In this by study, MR analyses were performed with the TwoSampleMR software package (version 0.5.6) and R software (version 4.2.1) (15). In this study, we used three analysis methods, namely, MR Egger, weighted median, and inverse variance–weighted (IVW), to determine whether there was a causal relationship between SLE and IBD. Of these, we used IVW as the primary method for MR analyses, which used weighted regression of SNP-specific Wald ratios to assess the causal effect of exposure on outcomes (16). MR Egger and weighted median were used as complementary analyses to test the robustness of the results: (1) weighted median (17): this method was allowed for consistent estimates of causal effects to be provided even when up to 50% of the IVs were invalidated. (2) MR Egger (18): the MR-Egger method was able to assess whether genetic variants have pleiotropic effects on the outcome and provides consistent estimates of causal effects under weaker assumptions, but the method might increase the type 1 error rate. Moreover, we used the MR-PRESSO method to identify anomalous outliers with horizontal pleiotropy (19).

### Sensitivity analysis

Several sensitivity analyses were used in this study to assess the robustness of the results. (1) Cochran’s Q test (20): the Cochran’s Q test assesses the heterogeneity among individual SNPs; if the P-value is greater than 0.05, then it is considered that there is no heterogeneity, in which case a fixed-effects IVW approach is used. If the P-value is less than 0.05, then the random effects IVW model is used. (2) MR Egger (21): this method was used to detect horizontal pleiotropy. (3) In addition, we used the leave-one-out method to determine whether the results of MR were significantly affected by any SNP.

## Results

### Selected genetic instruments (IVs)

We selected IVs according to the selection criteria described above. Consequently, 45 and 40 SNPs met the criteria as the IVs estimation of SLE in both Europe and East Asia, respectively (**Supplementary Tables**). The F-statistics were all greater than 10, and there was no evidence of weak instrumental bias (**Supplementary Tables**).

### Causal effects of SLE on IBD in a European population

**Figure 2:**
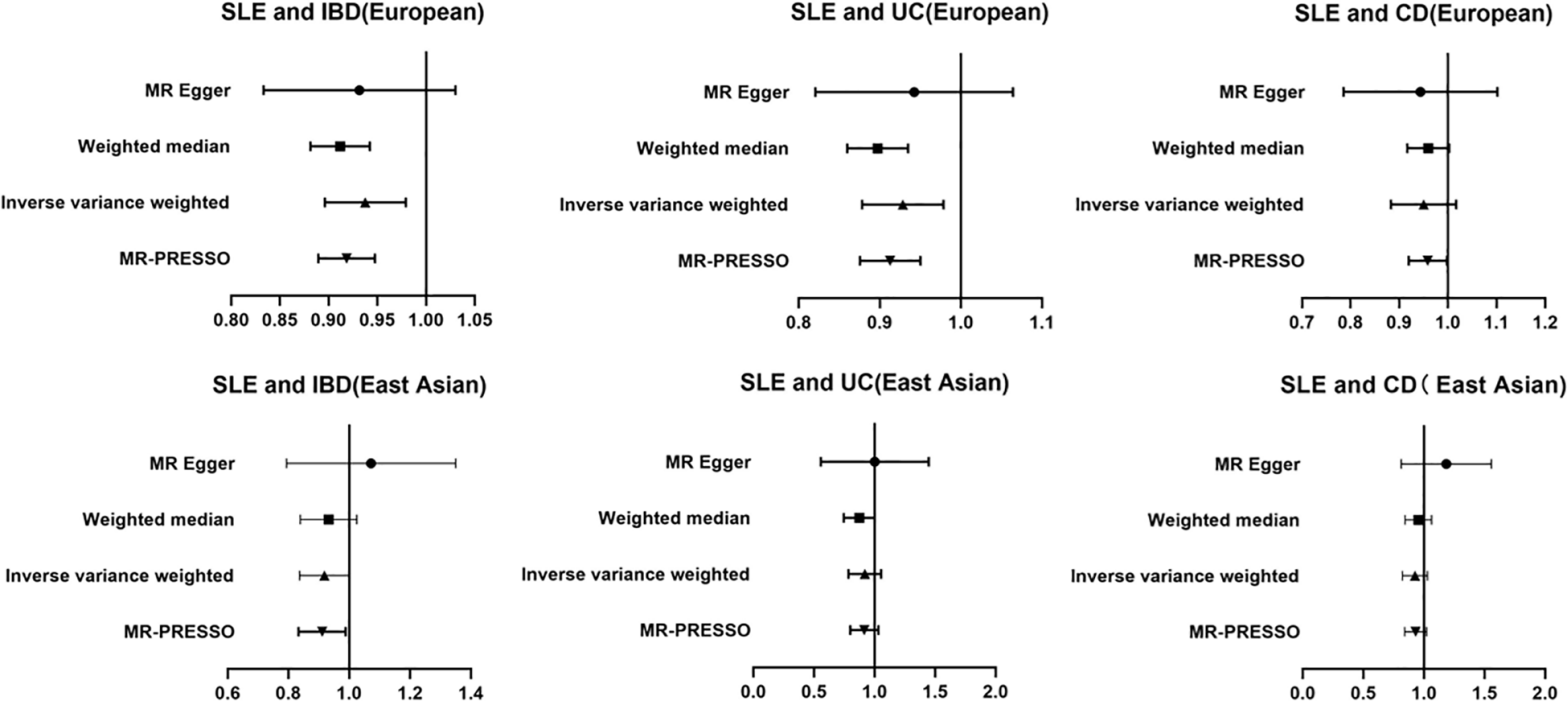
Forest plot of OR ratios for SLE and IBD among different racial populations.

**Figure 3:**
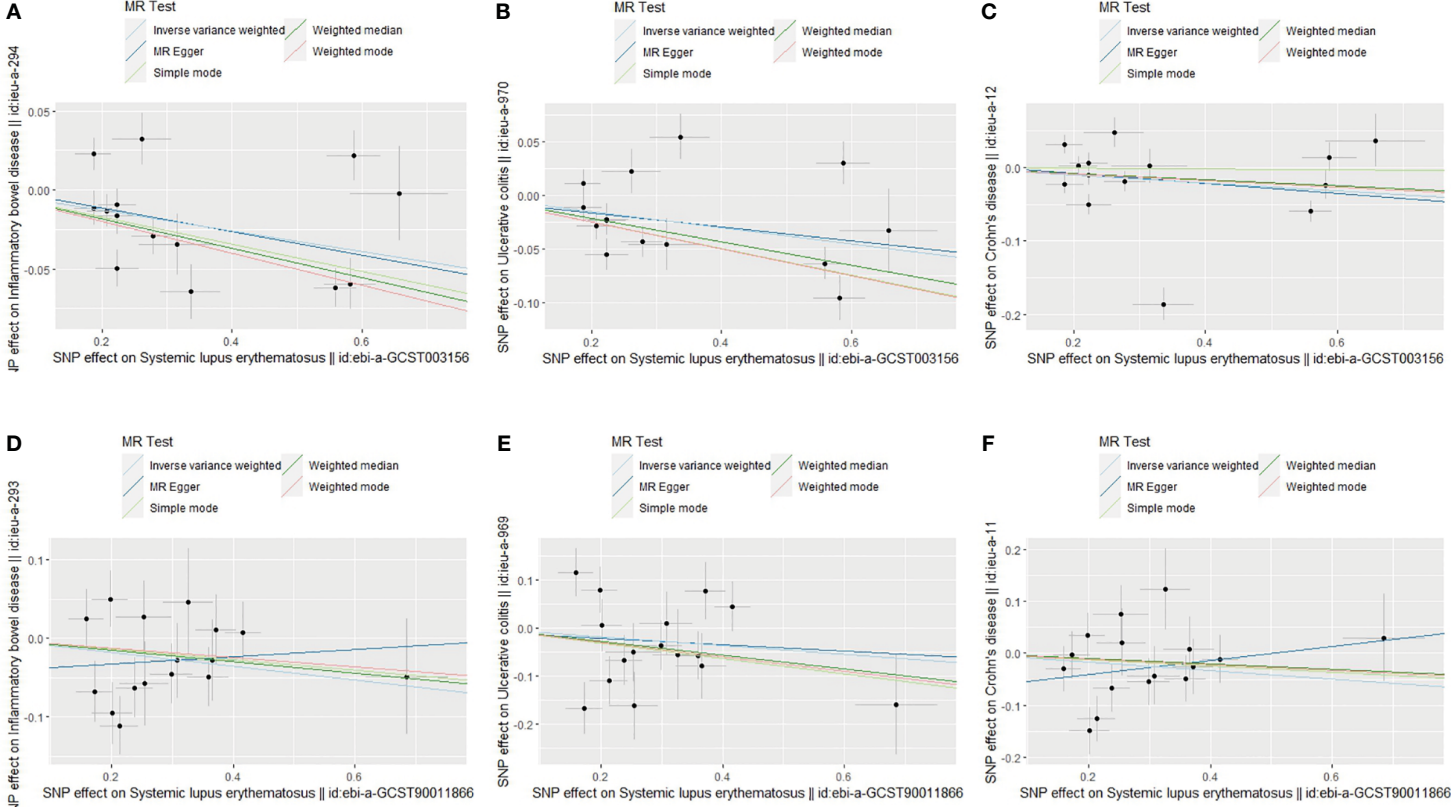
Scatter plot of the relationship between SLE and IBD. **(A)** SLE and overall IBD (Europeans); **(B)** SLE and UC (Europeans); **(C)** SLE and CD (Europeans); **(D)** SLE and overall IBD (East Asians); **(E)** SLE and UC (East Asians); **(F)** SLE and CD (East Asians).

The present study showed that SLE exhibited negative associations for all three outcomes (IBD, UC, and CD). Among them, there was a statistically significant association between SLE and overall IBD (OR = 0.94; 95% CI = 0.90, 0.98; P < 0.004) and UC (OR = 0.93; 95% CI = 0.88, 0.98; P = 0.006), respectively, and the results of the MR-PRESSO after removing the outliers were still in agreement with the IVW results. However, there was no statistically significant relationship between SLE and CD (OR = 0.95; 95% CI = 0.88, 1.02; P = 0.14) (**Figures 2**, **3**, **Table 2**).

**Table 2:** Results of MR analysis between SLE and IBD in both European and East Asian populations.

Outcome	Ancestor	Methods	OR	95% CI	*P*-value
IBD	European	MR-Egger	0.93	0.83–1.03	0.19
		Weighted median	0.91	0.88–0.94	<0.001
		IVW	0.94	0.90–0.98	0.004
		MR-PRESSO	0.92	0.89–0.95	<0.001
UC	European	MR-Egger	0.94	0.82–1.07	0.35
		Weighted median	0.90	0.86–0.94	<0.001
		IVW	0.93	0.88–0.98	0.006
		MR-PRESSO	0.91	0.88–0.95	0.002
CD	European	MR-Egger	0.93	0.79–1.11	0.45
		Weighted median	0.96	0.92–1.00	0.07
		IVW	0.95	0.88–1.02	0.14
		MR-PRESSO	0.96	0.92–1.00	0.06
IBD	East Asian	MR-Egger	1.05	0.81–1.36	0.73
		Weighted median	0.93	0.84–1.03	0.15
		IVW	0.92	0.84–1.00	0.052
		MR-PRESSO	0.91	0.83–0.99	0.04
UC	East Asian	MR-Egger	0.94	0.59–1.48	0.78
		Weighted median	0.87	0.75–1.00	0.06
		IVW	0.91	0.79–1.06	0.22
		MR-PRESSO	0.91	0.80–1.03	0.16
CD	East Asian	MR-Egger	1.15	0.83–1.6	0.42
		Weighted median	0.95	0.85–1.07	0.38
		IVW	0.92	0.82–1.03	0.15
		MR-PRESSO	0.93	0.84–1.02	0.15

SLE, systemic lupus erythematosus; IBD, inflammatory bowel disease; UC, ulcerative colitis; CD, Crohn’s disease.

### Causal effects of SLE on IBD in an East Asian population

Although SLE showed negative causal associations with overall IBD and UC in European populations, what is interesting was that, in this study, there was no statistically significant association between SLE and overall IBD (OR = 0.92; 95% CI = 0.84, 1.00; P = 0.052), UC (OR = 0.91; 95% CI = 0.79, 1.06; P = 0.22), and CD (OR = 0.92; 95% CI = 0.82, 1.03; P = 0.15) in the East Asian populations (**Figures 2**, **3**, **Table 2**).

### Sensitivity analysis of MR

First, in the heterogeneity test, the P-value of Cochran’s Q test was less than 0.05, indicating heterogeneity among SNPs (**Table 3**). Therefore, in this MR analysis, we used the random-effects IVW method as the main analysis method. MR Egger regression intercepts showed limited evidence of horizontal pleiotropy for SLE-related IVs. In addition, the leave-one-out method showed that the potential causal link between SLE and IBD in the European population was not driven by a SNP (**Supplementary Figure 4**). Moreover, forest and volcano plots provided a more visual display of heterogeneity (**Supplementary Figures 5**, **6**).

**Table 3:** Heterogeneity and horizontal pleiotropic test results.

Outcome	Pleiotropy test (MR-Egger)	SE	P	Heterogeneity test	Q_df	Q_pval	Ancestor
	Intercept			Q			
IBD	0.003	0.018	0.848	59.8	12	<0.001	European
UC	−0.003	0.021	0.865	56.2	12	<0.001	European
CD	0.005	0.028	0.853	103.9	12	<0.001	European
IBD	−0.041	0.039	0.302	21.6	14	0.088	East Asian
UC	−0.007	0.068	0.909	35.3	14	0.001	East Asian
CD	−0.07	0.048	0.176	23.4	14	0.05	East Asian

SLE, systemic lupus erythematosus; IBD, inflammatory bowel disease; UC, ulcerative colitis; CD, Crohn’s disease.

## Discussion

In the present study, two-sample MR study demonstrated that genetic susceptibility to SLE was causally associated with lower overall IBD risk and UC risk in European populations; however, no significant association between SLE and CD was found. Furthermore, interestingly, no association was found between SLE and IBD in East Asian populations. To the best of our knowledge, this is the first MR study to explore the causal relationship between SLE and IBD.

It is well known that SLE is considered an autoimmune disease, whereas previously, Mackay thought that IBD was not a strictly autoimmune disease because of the lack of specific serologic markers for IBD (22). However, in recent years, it has been found that the pathogenesis of IBD is inextricably linked to autoimmune factors (23). Recent studies have shown that the CXCL13/CXCR5 axis is activated in both SLE and IBD (24) and that its pathway is associated with the regulation of T cells. So far, the literature on the association between SLE and IBD consists mainly of a few case reports and case series (25), which seems to imply that the association between these two diseases is uncommon. It is worth noting, however, that the differentiation between SLE and IBD is sometimes difficult to make because SLE can also involve the gastrointestinal tract (25–27), and, thus, the association between the two diseases seems to be more than coincidental. In addition, it is noteworthy that patients with a combination of SLE and IBD do not have the perceived worse prognosis, and, on the contrary, patients tend to respond favorably to treatment (25, 28). To date, a relatively large database study has shown that the prevalence of CD is higher in patients with SLE (OR = 2.23; 95% CI = 1.46–3.4; P < 0.001) compared with controls, whereas SLE is not associated with UC (OR = 1.67; 95% CI = 0.99–2.815; P < 0.052) (29). This is contrary to our findings, which may be due to some confounding bias that is difficult to avoid, because people’s exposure to a risk factor may be related to a variety of factors, such as self-selection and occupation, and confounding bias exists when the given exposure is also closely related to another exposure that is associated with the outcome; studies based on management data may sometimes fail to accurately characterize the given exposures in each group, that is, population selection bias (30). In addition, the fact that the above study was conducted in a Middle Eastern population (Israelis), whereas the present study targeted European and East Asian populations, may also be a reason for the different conclusions, because the prevalence and pathogenesis of IBD may vary among different races and ethnic groups (31).

In this MR study, we confirmed a causal link between SLE and IBD in a European population. Previous studies have found that the *Homo sapiens* interferon regulatory factor 5 (IRF5) gene is strongly associated with the development of SLE (32), and a study from the United States suggests that polymorphisms in the IRF5 gene may be associated with protection against IBD (33); this is also consistent with our analysis (rs35000415, IRF5). In addition, further stratified analysis revealed an association between SLE and UC, but not between SLE and CD. Although there is an overlap in the pathogenesis of UC and CD, many recent studies have shown that they differ in genetics (34), pathogenesis (35, 36), cellular immunity (37), and response to probiotic therapies (38), which may explain the absence of a causal link between SLE and CD. However, further studies are needed to investigate the similarities and differences in genetics, intestinal flora, and pathogenesis between the two diseases. Talking about East Asian populations, no association was found between SLE and IBD, which might be due to the differences in the genes associated with SLE (39) and IBD (40) in East Asian populations compared with European populations. For example, Asian patients with IBD are less likely to carry mutants in the nucleotide-binding oligomerization domain 2 (NOD2) gene compared with Europeans (11), and studies have shown that NOD2 is strongly associated with the development of SLE (41). However, although there was no statistically significant difference between SLE and IBD in the East Asian population (P = 0.052), the P-value was very close to 0.05, which might be related to the small sample volume of the East Asian population, and, thus, a larger sample size is needed to further investigate the relationship between the two.

Our study had several strengths. First, we used MR to assess the association between SLE and IBD, which was less susceptible to confounders, reverse causation, etc., than observational studies (42). Second, our exposed IVs were derived from large-scale GWAS, which provided robust and reliable gene-wide associated SNP associations, avoiding bias caused by weak instruments. In addition, we used the MR-PRESSO method to further confirm the reliability of this study.

However, there are some limitations to our study. First, although we used multiple methods to analyze horizontal pleiotropy and the results were consistent across multiple methods of analysis, there was still no guarantee that potential horizontal pleiotropy was ruled out completely. Second, the findings of this study could not be extrapolated to other races due to the racial limitations of this study. In addition, because of the limitations of sample volume and gene sequencing technology, we look forward to further exploring the association between SLE and IBD in larger-scale research in the future.

## Conclusion

This MR work revealed a negative causal effect of SLE on overall IBD and UC in European populations, but not between SLE and CD. In contrast, there was no causal relationship between SLE and IBD in East Asian populations. Our results may enrich previous studies and may provide a reference for future animal experiments and clinical treatments.

## Data availability statement

The original contributions presented in the study are included in the article/**Supplementary Material**. Further inquiries can be directed to the corresponding authors.

## Ethics statement

Ethical approval was not required for the study involving humans in accordance with the local legislation and institutional requirements. Written informed consent to participate in this study was not required from the participants or the participants’ legal guardians/next of kin in accordance with the national legislation and the institutional requirements.

## Author contributions

WX completed data analysis and most of the manuscript writing. NL and SH provided constructive comments and pertinent revisions to the manuscript. HZ, YS, ZY, GH, and YC completed the data collection. HJ, HX, and SH completed the data verification and checked the grammar of the article. SL provided constructive suggestions on the logic, grammar, and other issues of the article during the revision stage. In addition, he also provided meaningful suggestions on how to conduct further in-depth research in this field in the future. In addition, this article later received support from SL’s related funds: Natural Science Foundation of Zhejiang Province (LQ20H020002). All authors contributed to the article and approved the submitted version.
