# Supplementary material for: Association between systemic lupus erythematosus and inflammatory bowel disease in European and East Asian populations: a two-sample Mendelian randomization study
Source: Front Immunol. 2023 Nov 3;14:1199896. doi: 10.3389/fimmu.2023.1199896 (PMC10654968; doi:10.3389/fimmu.2023.1199896)
Supplement: Supplementary file 1 [file DataSheet_1.docx]

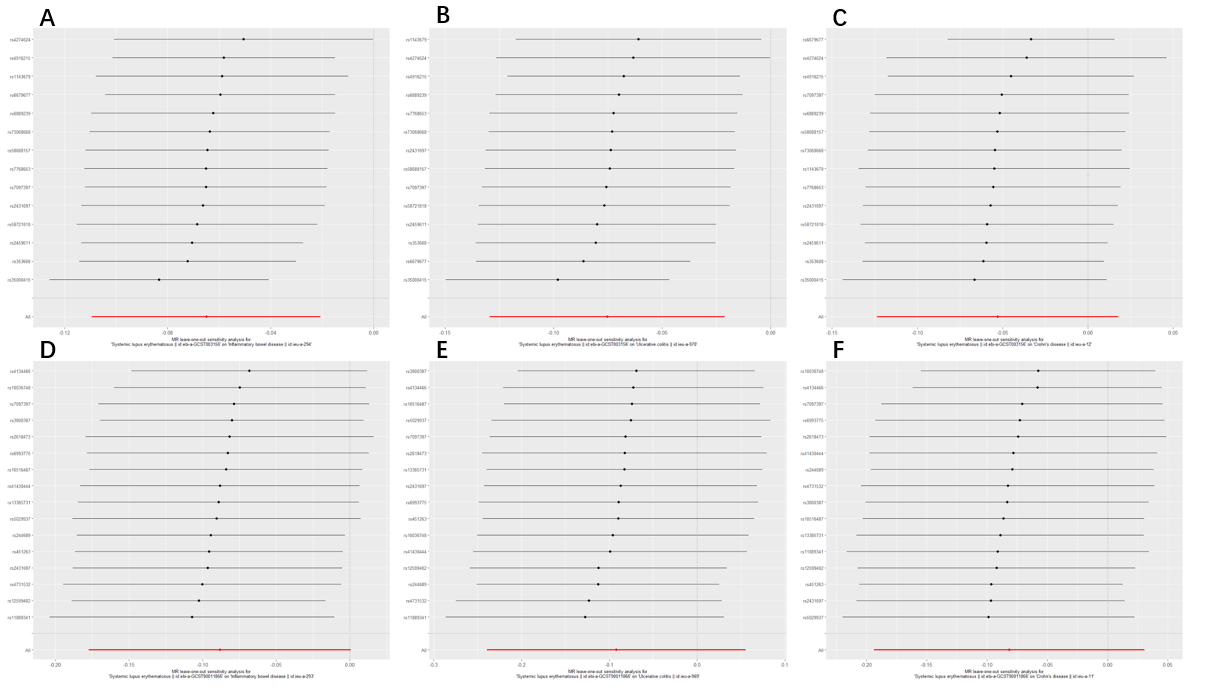


Figure 4: Leave-one-out sensitivity test

A: SLE and overall IBD (Europeans); B: SLE and UC (Europeans); C: SLE and CD (Europeans); D: SLE and overall IBD (East Asians); E: SLE and UC (East Asians ); F: SLE and CD (East Asians)


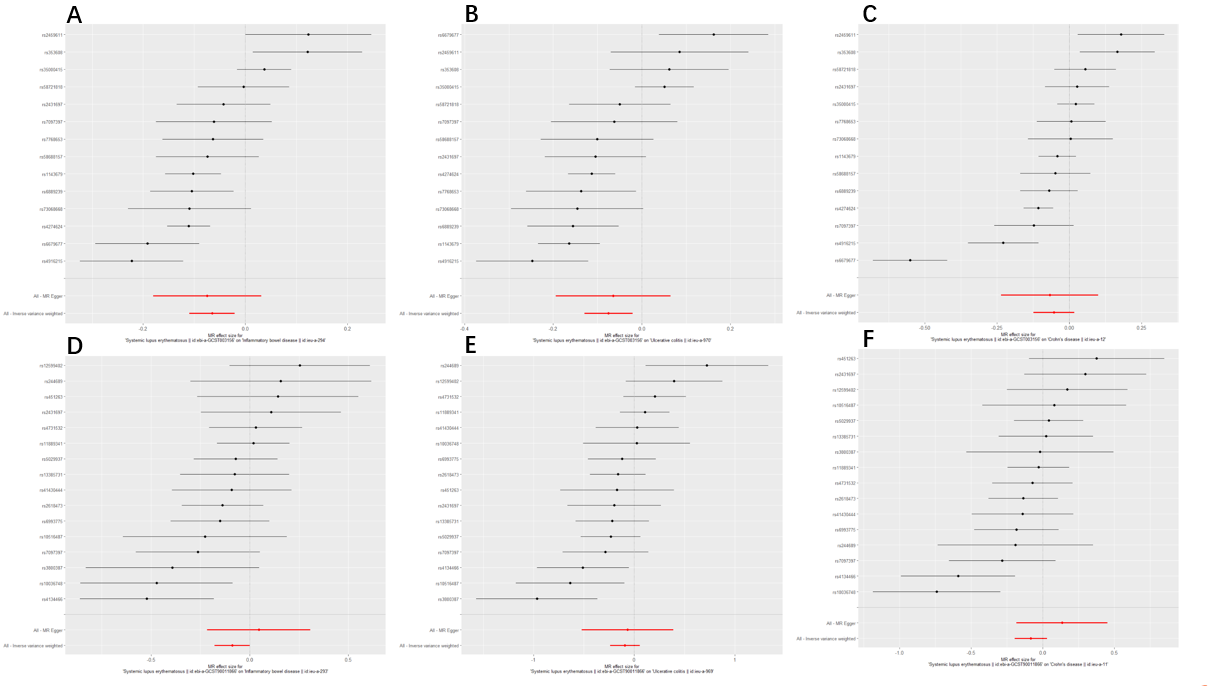


Figure 5: Forest plot of the relationship between SLE and IBD

A: SLE and overall IBD (Europeans); B: SLE and UC (Europeans); C: SLE and CD (Europeans); D: SLE and overall IBD (East Asians); E: SLE and UC (East Asians ); F: SLE and CD (East Asians)


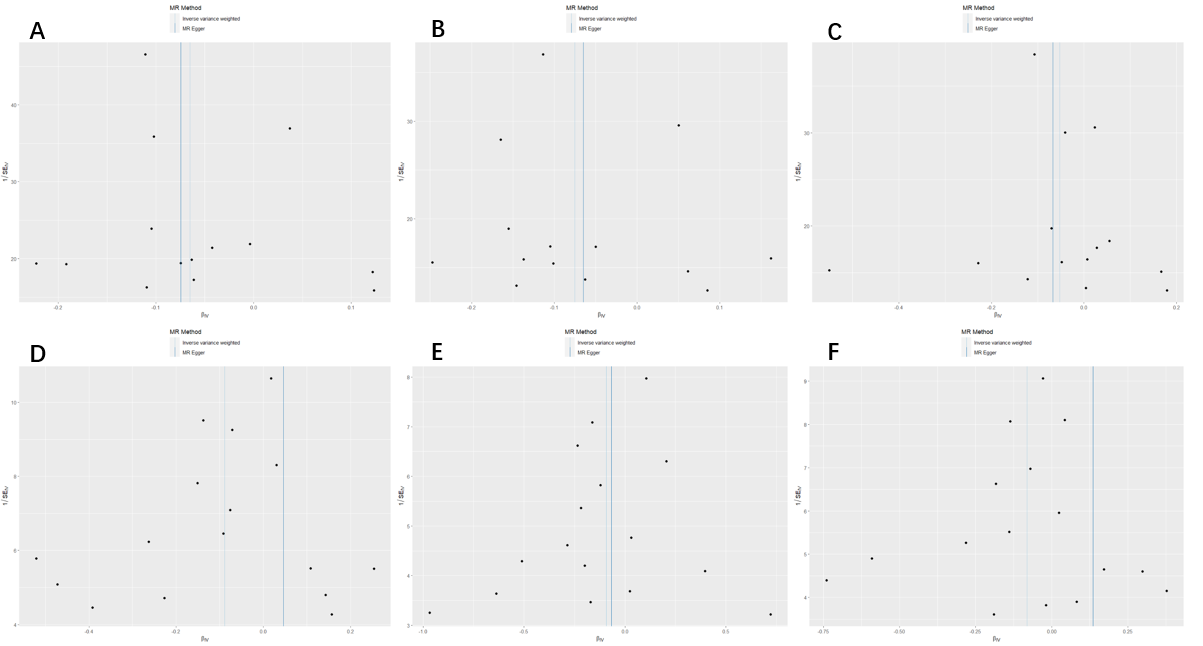


Figure 6: Volcano plot of MR results

A: SLE and overall IBD (Europeans); B: SLE and UC (Europeans); C: SLE and CD (Europeans); D: SLE and overall IBD (East Asians); E: SLE and UC (East Asians ); F: SLE and CD (East Asians)
